# Inhibitory effect of endostar combined with radiotherapy on gastric cancer animal models

**DOI:** 10.1186/s12957-020-01937-1

**Published:** 2020-07-15

**Authors:** Qitian Chen, Ran Chen, Youhong Dong

**Affiliations:** Department of Oncology, Xiangyang No. 1 People’s Hospital, Hubei University of Medicine, 15 Jiefang Road, Xiangyang, 441000 Hubei People’s Republic of China

**Keywords:** Endostar, Gastric cancer animal model, Interleukin-10, Radiotherapy, Transforming growth factor-β1

## Abstract

**Background:**

Inhibitory effect of endostar combined with radiotherapy on gastric cancer (GC) animal models and its effect on transforming growth factor-β1 (TGF-β1) and inter-leukin-10 (IL-10) were evaluated.

**Methods:**

Forty mice of GC model xenograft tumors were prepared and randomly divided into blank control group, endostar group, radiotherapy group, and endostar combined with radiotherapy group (combination group). From the 14th day, a vernier caliper was used for measuring the long and short diameters of the xenograft tumors. The formula V = ab^2^/2 was used for calculating the tumor volume and to obtain its average value. Tumor growth curves were plotted to calculate the tumor inhibition rate. The growth of xenograft tumors and the behavioral changes of mice were observed. Enzyme-linked immunosorbent assay (ELISA) was used for detecting the expression levels of IL-10 and TGF-β1.

**Results:**

The tumor growth in the combination group was significantly inhibited, and the tumor volume was the smallest compared with the other groups (*p* < 0.05). Compared to the blank control group, the tumor inhibition rate was 11.8% in endostar group, 33.0% in radiotherapy group, and 52.1% in combination group (*p* < 0.01). Endostar combined with radiotherapy had an interaction in decreasing the expression levels of TGF-β1 and IL-10 (*F* = 4.35 and 5.12, *p* < 0.05). Leucocyte count was significantly higher in control and combination groups than that in endostar and radiotherapy groups. The body weight of mice in endostar and radiotherapy groups decreased after treatment (*p* < 0.05). The body weight of mice after treatment in control and combination groups increased, with a statistically significant difference compared to that before treatment (*p* < 0.05). There was a statistically significant difference among all groups after treatment (*F* = 198.1, *p* < 0.01).

**Conclusions:**

Endostar combined with radiotherapy can inhibit tumor growth and downregulate the expression levels of TGF-β1 and IL-10 through synergistic action.

## Background

Gastric cancer (GC) is one of the most common malignant tumors in the world [1]. Tumor cells that grow like normal cells require blood to provide nutrition and oxygen; however, malignant tumors produce macronutrients and growth factors that cause neovascularization in tumor tissues to grow more than that in normal tissues, leading to uncontrolled tumor growth. Therefore, controlling tumor angiogenesis is an effective method for treating tumors [2, 3]. Palliative radiotherapy and chemotherapy are commonly used in the treatment of gastric cancer, which can prolong the survival time of patients and improve the quality of life [4]. Palliative radiotherapy and chemotherapy have therapeutic effects that are minimal however. In recent years, as molecular biology develops, molecular targeted therapy has become a new antitumor treatment. Studies have shown that anti-angiogenesis drugs have achieved good effects on the comprehensive treatment of GC. However, treatment with anti-angiogenesis drugs alone cannot cure tumors. Recombinant human endostatin (endostar), a broad-spectrum anti-angiogenesis drug, has synergistic antitumor effects in combination with radiotherapy [5–7]. However, there are few reports on whether endostar combined with radiotherapy can inhibit tumor lymphangiogenesis. In this study, the inhibitory effect of endostar combined with radiotherapy on GC cells in mice was investigated.

Transforming growth factor-β (TGF-β) superfamily is one of the factors regulating tumor angiogenesis [8]. TGF-β is a multifunctional cytokine produced mainly by cell autocrine and paracrine. The main role of TGF-β1 is to stimulate proliferation, differentiation, and migration of cells, as well as the chemotaxis of inflammatory cells, thereby affecting angiogenesis, controlling the synthesis and degradation of extracellular matrix [9]. TGF-β1 also has an immunosuppressive effect. It can promote tumor invasion and metastasis in vivo by inhibiting the activity of natural killer (NK) cells in the body, the expression of which is closely related to malignant biological behaviors of cancer cells in GC, such as invasion and metastasis [10]. Interleukin-10 (IL-10) is an immune factor located in the immune regulatory network center, with a dual role of immune stimulation and inhibition [11]. IL-10 gene has been confirmed to be one of the genes related to GC. IL-10 can promote the occurrence of GC mainly due to the combination of gene status changes and environmental factors. IL-10 gene promoter is responsible for binding its base changes to gene expression regulators, directly affecting gene transcription and expression and thus gene transcriptional activity. The high expression of IL-10 gene can cause immune responses and specific inhibition by acting on a series of signal transduction systems, further inhibiting multiple biological effects of the body [12].

In this study, the inhibitory effect of endostar combined with radiotherapy on cancer cells in GC animal models and its effect on the expression of TGF-β1 and IL-10 were studied, to explore the inhibitory effect of endostar combined with radiotherapy on GC, providing a reference for future clinical experiments.

## Methods

### Animals

A total of 40 female mice were selected, aged 6–8 weeks, with a body mass of 18–20 g, purchased from Hunan slack Jingda Experimental animal Co., Ltd. During the experiment, all raw materials, feed, and drinking water were disinfected at super pressure-high temperature and dried in an oven at 180 °C. Mice were fed in a specific pathogen-free (sPF) barrier system, had free access to food and water, and were kept at 23–25 °C, on a 12 h light/12 h dark cycle, and humidity 55 ± 10%. The study was approved by the Ethics Committee of Xiangyang No. 1 People’s Hospital (Xiangyang, China). The experimental procedures were in accordance with the Animal Research: Reporting of In Vivo Experiments (ARRIVE) guidelines.

### Tumor model establishment

Mouse tumor models were established. Mouse GC cells (MFC; cat. no. BNCC100207) in logarithmic growth phase were purchased from BeNa Culture Collection. Saline was used for dilution to prepare the cell suspension, the concentration of which was adjusted to ~ 10^10^/l. The left abdomen skin of the mice was disinfected with iodophor, and 0.2 ml of cell suspension was inoculated in mice [13]. The needle was kept as oblique as possible during the operation. After experimental inoculation, mice were immediately returned to their original place to continue feeding, if their behavioral state was normal. Tumors formed after ~ 1 week (the tumor nodule was approximately 8–10 mm in diameter). Then, the growth of xenograft tumors in mice was examined on the 2nd day. The behavioral changes of the mice during the experiment were observed and recorded, mainly in terms of activity, body mass, mental state, eating, and drinking, with timely measures taken. Vernier caliper was used for measuring the long and short diameters of xenograft tumors, and the tumor volumes were calculated.

### Experimental grouping and treatment measures

When the xenograft tumor volume in mice reached 8–10 mm^3^, the 40 tumor-bearing mice were randomly divided into control group, endostar group, radiotherapy group, and endostar combined with radiotherapy group (referred to as combination group); 10 mice in each group. There was no statistically significant difference in the body mass of mice among the groups before the experiment (*p* > 0.05). (i) In control group, saline (0.2 ml/day) was injected through the vena caudalis for 14 consecutive days. (ii) In endostar group, endostar (Shandong Xiansheng Maidejin biological Pharmaceutical Co., Ltd.; sFDa approval no. s20050088) of 5 mg/kg was injected through the vena caudalis, with each ~ 0.2 ml, for 14 consecutive days. (iii) In radiotherapy group, saline (0.2 ml/day) was injected through the vena caudalis for 14 consecutive days. On the 7th day of the experiment, 1% chloral hydrate (Qingdao Yulong Seaweed Co., Ltd.; SFDA approval no. H37022673) was used for anesthesia in mice, which were fixed in appropriate body position. At the same time, mice were covered with tissue equivalent materials (~ 1 cm in thickness) and irradiated with 6 MV X-rays. The total dose of radiotherapy in the experiment was 10 Gy, and the source-skin distance was 100 cm, for 1 day of radiotherapy. (iv) In combination group, endostar (5 mg/kg) was injected through the vena caudalis for 14 consecutive days, ~ 0.2 ml/mouse. Radiotherapy was given on the 7th day of administration, with the same specific treatment measures as those in the radiotherapy group.

At the same time, the growth of xenograft tumors and behavioral changes of mice in each group were observed. Mice were sacrificed by cervical dislocation at 24 h after the last experimental operation. Their tumor tissues were removed and weighed with an electronic balance after washing with saline. Filter paper was used to absorb moisture. Then, these tumor tissues were numbered and recorded and stored in a freezer at − 80 °C.

### Observation indicators

(i) The growth of xenograft tumors in each group of mice was observed. (ii) The behavioral state, such as mental state, eating, drinking, activity, and body weight, was observed. The vernier caliper was used for measuring (a) the long diameter and (b) short diameter of the xenograft tumors every 3 days, and the values were recorded. The formula V = ab^2^/2 was used for calculating the tumor volume, and the average value was also calculated for plotting the tumor growth curve of mice in each group. (iii) The tumor mass was weighed with an electronic balance, and the related formula was used to obtain the tumor growth inhibition rate (IR). Formula IR = [1 − average tumor mass in experimental group (endostar group, radiotherapy group, or combination group)/average tumor mass in blank control group] × 100%.

### Detection of hematology and serum protein levels

Blood was taken by eyeball removal method at 24 h after the last administration. The leucocyte count, erythrocyte count, platelet count, and expression levels of serum total protein and serum albumin in each group were detected.

### Detection of TGF-β1 and IL-10 expression in the supernatant of tumor tissues

Enzyme-linked immunosorbent assay (ELISA) was used to determine the serum TGF-β1 and IL-10 levels. TGF-β1 and IL-10 kits were provided by Moshake Biotechnology Co., Ltd. (item nos. 69-36961 and 69-99847, respectively). The instrument was BS-1101 micro-plate reader from Beijing Linmao Technology Co., Ltd. All operations were strictly in accordance with the manufacturer’s instructions.

### Statistical analysis

The SPSS 17.0 statistical software (Tianjin Network Technology Co., Ltd.) was used to statistically analyze the experimental data. Measurement data were expressed as mean ± SD. The analysis of variance of two-factor factorial design was used for the comparison of the expression levels of TGF-β1 and IL-10 among multiple groups, one-way analysis of variance for the comparison of the measurement data of hematology, and serum protein level among multiple groups, and LSD *t* test was the post hoc test. *t* test was used for the comparison of body weight before and after treatment in the same group. *p* < 0.05 was considered to indicate a statistically significant difference.

## Results

### Comparison of tumor growth among the four groups of mice

**Fig. 1:**
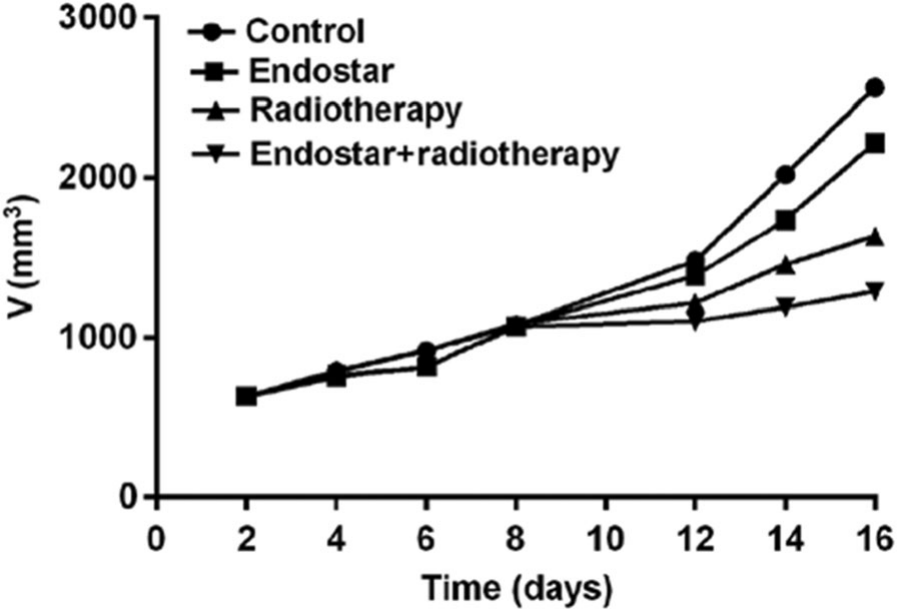
Comparison of tumor growth among the four groups of mice. The tumor growth in endostar and combination groups was slow and was significantly inhibited in the combination group after the 7th day of local radiotherapy. Before radiotherapy, the tumor growth rate in the radiotherapy group was equivalent to that in the control group. After radiotherapy, the tumor growth gradually became slower, and the tumor volume in radiotherapy group was finally smaller than that in endostar and control groups. As can be seen from the curves, in the combination group of mice, the tumor growth rate was the slowest, the tumor volume was the smallest, and the antitumor effect was the most significant (*p* < 0.05)

After the experiment, the tumor growth curves of mice were plotted (Fig. 1). As can be seen, the tumor growth in endostar and combination groups was slow. After the 7th day of local radiotherapy, the tumor growth was significantly inhibited in the combination group that was finally the slowest, and the tumor volume was the smallest compared to that of the other groups. Before radiotherapy, the tumor growth rate and the tumor volume in radiotherapy group were not much different from those in control group. When radiotherapy group received radiotherapy on the 7th day of the experiment, the tumor growth rate gradually became slower, and the tumor volume was finally smaller than that in endostar and control groups. The final tumor volumes of mice in control, endostar, radiotherapy, and combination groups were 2992 ± 139.12, 2341 ± 768.02, 1967.26 ± 612.23, and 1645 ± 327.1 mm^3^, respectively. The antitumor effect in the combination group was the most significant, with statistically significant differences among groups (*p* < 0.05).

### Comparison of tumor mass and inhibition rate among the four groups.

The tumor mass in control, endostar, radiotherapy, and combination group was 3.30 ± 0.31, 2.91 ± 0.19, 2.21 ± 0.14, and 1.58 ± 0.15 g, respectively (Table 1). The tumor inhibition rate (%) was calculated according to the tumor mass of the four groups. Compared to blank control group, the tumor inhibition rate was 11.8% in endostar group, 33.0% in radiotherapy group, and 52.1% in combination group. There was a significant difference in tumor weight between the four groups. The tumor weight of the control group was higher than that of the other three groups. The tumor weight of the combination group was significantly lower than that of the endostar group and the radiotherapy group. The difference was statistically significant (*p* < 0.01).

**Table 1:** Comparison of tumor mass and inhibition rate among the four groups (mean ± SD)

Group	No. of mice	Tumor mass (g)	Inhibition rate (%)
Control	10	3.30 ± 0.31	-
Endostar	10	2.91 ± 0.19^a^	11.8
Radiotherapy	10	2.21 ± 0.14^a,b^	33.0
Combination	10	1.58 ± 0.15^a–c^	52.1
*F* value		133	
*p* value		< 0.01	

^a^*p* < 0.01 compared to control group

^b^*p* < 0.01 compared to endostar group

^c^*p* < 0.01 compared to radiotherapy group

### Comparison of TGF-β1 expression in the supernatant of tumor tissues among the four groups

The variance results of factorial design showed that endostar combined with radiotherapy had an interaction in decreasing the expression level of TGF-β1 (*F* = 4.35, *p* < 0.05), indicating a synergistic action between endostar and radiotherapy (Table 2). Regardless of whether radiotherapy was used or not, endostar reduced the TGF-β1 content in tumor tissues (*p* < 0.05). The TGF-β1 content was significantly lower in endostar group than that in control group (*p* < 0.05). There was a statistically significant difference between the radiotherapy and the control group (*p* < 0.05). The relative expression of TGF-β1 was significantly lower in combination group than that in radiotherapy group (*p* < 0.05). The TGF-β1 content in combination group was the lowest, with the best inhibitory effect (Table 2).

**Table 2:** Comparison of TGF-β1 expression in the supernatant of tumor tissues among the four groups (mean ± SD)

Radiotherapy				
Endostar	+	−	*t* value	*p* value
+	101.14 ± 56.03	174.76 ± 55.35	2.975	< 0.05
−	226.80 ± 53.89	279.01 ± 56.02	2.110	< 0.05
*t* value	5.078	4.213		
*p* value	< 0.05	< 0.05		

*TGF-β1* transforming growth factor-β1

### Comparison of IL-10 expression in the supernatant of tumor tissues among the four groups

The variance results of factorial design showed that endostar combined with radiotherapy had an interaction in decreasing the expression level of IL-10 (*F* = 5.12, *p* < 0.05), indicating a synergistic action between endostar and radiotherapy (Table 3). Regardless of whether radiotherapy was used or not, endostar reduced the IL-10 content in tumor tissues (*p* < 0.05). The IL-10 content was significantly lower in endostar group than that in control group (*p* < 0.05). There was a statistically significant difference between the radiotherapy and the control group (*p* < 0.05). The relative expression of IL-10 was significantly lower in combination group than that in radiotherapy group (*p* < 0.05). The IL-10 content was the lowest in combination group, with the best inhibitory effect (Table 3).

**Table 3:** Comparison of IL-10 expression in the supernatant of tumor tissues among the four groups (mean ± SD)

Radiotherapy				
Endostar	+	−	*t* value	*p* value
+	81.43 ± 28.14	132.62 ± 31.12	2.858	< 0.05
−	152.60 ± 39.09	189.67 ± 55.91	2.070	< 0.05

*IL-10* interleukin-10

### Detection results of hematology and serum protein levels after the experiment in each group of mice

The results of one-way analysis of variance showed that there was no statistically significant difference in leucocyte count between the endostar and the radiotherapy group (*p* > 0.05) or between the control and the combination group (*p* > 0.05), but leucocyte count was significantly higher in control and combination groups than that in endostar and radiotherapy groups (*p* < 0.05) (Table 4). There were no statistically significant differences in erythrocyte and platelet counts among all groups, neither in total protein and albumin contents among the groups (*p* > 0.05) (Table 4).

**Table 4:** Results of hematology and serum protein levels after the experiment in each group of mice (mean ± SD)

Group	No. of cases	Leucocyte count (× 109·l−1)	Erythrocyte count (× 1012·l−1)	Platelet count (× 109·l−1)	Total protein content (g·l−1)	Albumin content (g·l−1)
Control	10	4.93 ± 1.63	9.41 ± 0.43	494.0 ± 57.12	43.7 ± 2.65	15.3 ± 1.24
Endostar	10	1.64 ± 0.68a	8.87 ± 1.02	487.1 ± 61.3	42.7 ± 1.87	16.1 ± 2.31
Radiotherapy	10	1.78 ± 0.72a	8.68 ± 1.24	472.3 ± 70.2	43.5 ± 2.42	15.9 ± 1.65
Combination	10	3.87 ± 1.24b,c	9.26 ± 0.65	498.6 ± 79.4	43.1 ± 2.31	15.1 ± 1.43
*F* value		20.60	1.44	0.29	0.36	0.78
*p* value		< 0.05	0.25	0.83	0.78	0.51

^a^*p* < 0.05 compared to control group

^b^*p* < 0.05 compared to endostar group

^c^*p* < 0.05 compared to radiotherapy group

### Adverse reactions and body weight changes during treatment

After treatment, the mice in endostar and radiotherapy groups continuously showed low spirit, slow response, decreased activity, decreased eating to different degrees, and decreased body weight caused by diarrhea. However, the mice in combination and control groups showed good mental state, normal activity, rapid response, normal eating and drinking, and normal urination or defecation (Table 5). Mice were weighed before sacrificed: the body weight of mice in endostar and radiotherapy groups decreased. The body weight was 13.89 ± 0.26 g in endostar group and 13.41 ± 0.28 g in radiotherapy group, with a statistically significant difference between before treatment and after treatment (*p* < 0.05). The body weight of mice after treatment in control and combination groups increased, with a statistically significant difference between before treatment and after treatment (*p* < 0.05). There was no statistically significant difference among the four groups before treatment (*p* > 0.05), but a statistically significant difference was observed after treatment (*F* = 198.1, *p* < 0.01).

**Table 5:** Comparison of body weight changes before and after treatment in each group (mean ± SD)

Group	Body weight before treatment (g)	Body weight after treatment (g)	*t* value	*p* value
Control	16.76 ± 0.91	16.80 ± 0.52	− 0.345	0.016
Endostar	16.82 ± 0.93	13.89 ± 0.26^a^	9.503	0.035
Radiotherapy	16.74 ± 0.92	13.41 ± 0.28^a^	12.70	< 0.01
Combination	17.01 ± 1.13	18.34 ± 0.84^a–c^	− 6.135	0.018

^a^*p* < 0.05 compared to the body weight after treatment in control group

^b^*p* < 0.05 compared to the body weight after treatment in endostar group

^c^*p* < 0.05 compared to the body weight after treatment in radiotherapy group

## Discussion

GC has high incidence and mortality, ranking 4th and 3rd in malignant tumors, respectively [14]. Many patients are already in advanced stage when diagnosed with GC. At present, chemotherapy is widely used in patients with advanced GC as a main treatment for tumors [15]. Although it prolongs the survival time of patients to a certain extent, it often causes severe adverse reactions during the treatment, which some patients are intolerant to [15]. With the continuous deepening of research on GC, many new treatments have begun to be applied clinically and achieve good efficacy. The molecular targeted therapy is one of them. Endostar, as an endostatin, is an endogenous angiogenesis inhibitor. Studies have found that by binding to multiple targets, it blocks pathological angiogenesis and changes the tumor microenvironment, inhibiting tumor formation and metastasis [16]. Studies have shown that the efficacy of endostar combined with radiotherapy and chemotherapy has a great application prospect [17, 18]. In this study, endostar combined with radiotherapy had a more significant inhibitory effect on mouse tumors.

Overexpression of TGF-β1 is common in malignant tumor tissues, closely related to the occurrence, development, and prognosis of tumors [19–22]. In the early stage of tumors, TGF-β1 inhibits tumor growth. However, in the rapid development stage, it promotes the interaction of tumor cells with extracellular matrix and tumor neovascularization, playing an immunosuppressive role, thereby accelerating the occurrence, development, and metastasis of tumors [23]. As a cytokine, IL-10 is important in immune modulatory responses secreted by macrophages, with a positive immune modulatory effect and a negative immunosuppressive effect [24]. It is an immunosuppressive factor that can inhibit the expression of histocompatibility complex (MHCII)-like molecules on the surface of antigen-presenting cells, thereby inhibiting its antigen ability. IL-10 can inhibit cytotoxicity mediated by helper T cells and NK cells and stimulate tumor immune tolerance, thereby inhibiting antitumor immune responses and accelerating tumor progression [25].

In this study, mouse models of GC xenograft tumors were established, to compare the tumor growth among groups and calculate the tumor inhibition rate. The results showed that the tumor growth volume of mice in endostar combined with radiotherapy group was the smallest, and the tumor inhibition rate was the highest, reaching 52.1%. This further confirms that endostar combined with radiotherapy has a synergistic action on the treatment of GC [5–7]. In this study, the variance of factorial design revealed that the expression of TGF-β1 and IL-10 in the supernatant of mouse tumor tissues decreased after treatment with endostar and radiotherapy, with statistically significant differences (*p* < 0.05). Their relative expression was lower after endostar combined with radiotherapy treatment than that of endostar or radiotherapy alone. By relatively analyzing the expression of TGF-β1 and IL-10, it is further concluded that endostar and radiotherapy can play a synergistic role. This is mainly achieved by increasing the sensitivity of radiotherapy through endostar, but the specific mechanism is not very clear at the moment. According to the existing literature, the mechanism may be as follows: (i) immature tumor blood vessels are corrected to restore normalization, improving the hypoxic state in tumors [26, 27]. (ii) The cell cycle is adjusted, and endostar can arrest most cells in G2/M phase that is the most sensitive to radiotherapy [28]. (iii) It has a certain effect on endothelial cells, which inhibits the tumor growth by inhibiting the migration, growth, and apoptosis of endothelial cells in the body [29, 30].

In this study, the hematology and serum protein levels of mice after the experiment in each group were detected. The results showed that leucocyte count was significantly higher in control and combination groups than that in endostar and radiotherapy groups, but there was no difference among the other groups. It is suggested that endostar combined with radiotherapy can reduce hematotoxicity. The specific mechanism currently lacks relevant literature and needs further research. In this study, adverse reactions and body weight were also analyzed during the treatment. The results showed that adverse reactions of endostar and radiotherapy alone were more severe than those in combination and control groups, and the body weight decreased. It showed that endostar combined with radiotherapy can reduce the incidence of adverse reactions of endostar or radiotherapy alone. This is related to their synergistic action on inhibiting tumor growth and development. However, there is still no relevant literature on the specific mechanism, which needs to be confirmed by further experiments.

In the present study, tumor growth, tumor mass, tumor inhibition rate, and expression of TGF-β1 and IL-10 in the supernatant of tumor tissues of mice were compared among groups; the hematology and serum protein levels of mice after the experiment were detected, and adverse reactions and changes in body weight during the treatment were studied. However, the microbial environment in GC models was not examined. Moreover, only the cytokines TGF-β1 and IL-10 were tested, and many cellular immune cells were not monitored. Thus, further study is needed.

## Conclusions

Endostar combined with radiotherapy can inhibit the tumor growth and downregulate the expression levels of TGF-β1 and IL-10 through a synergistic action, providing further reference for later clinical experiments.

## Data Availability

The datasets used and/or analyzed during the current study are available from the corresponding author on reasonable request.
